# Effect of Kidney Transplantation on Accelerated Immunosenescence and Vascular Changes Induced by Chronic Kidney Disease

**DOI:** 10.3389/fmed.2021.705159

**Published:** 2021-09-27

**Authors:** Noemi Ceprian, Gemma Valera, Jara Caro, Claudia Yuste, Nadia Serroukh, Ignacio González de Pablos, Carlos Oliva, Andrea Figuer, Manuel Praga, Matilde Alique, Rafael Ramirez, Enrique Morales, Julia Carracedo

**Affiliations:** ^1^Departamento de Genética, Fisiología y Microbiología, Universidad Complutense de Madrid, Instituto de Investigacin Sanitaria Hospital 12 de Octubre (imas12), Madrid, Spain; ^2^Departamento Biología de Sistemas (Unidad Fisiología), Facultad de Medicina, Instituto Ramón y Cajal de Investigación Sanitaria (IRYCIS), Alcalá de Henares, Madrid, Spain; ^3^Departamento de Nefrología, Hospital Universitario 12 de Octubre, Instituto de Investigación Sanitaria 12 de Octubre, Madrid, Spain; ^4^Departamento de Nefrología, Hospital Universitario 12 de Octubre, Madrid, Spain; ^5^Departamento de Genética, Fisiología y Microbiología, Universidad Complutense de Madrid, Madrid, Spain

**Keywords:** chronic kidney disease, immunity, immunosenescence, microvesicles, renal transplantation

## Abstract

Kidney transplantation is the best option for patients with end-stage renal disease. Despite the improvement in cardiovascular burden (leading cause of mortality among patients with chronic kidney disease), cardiovascular adverse outcomes related to the inflammatory process remain a problem. Thus, the aim of the present study was to characterize the immune profile and microvesicles of patients who underwent transplantation. We investigated the lymphocyte phenotype (CD3, CD4, CD8, CD19, and CD56) and monocyte phenotype (CD14, CD16, CD86, and CD54) in peripheral blood, and endothelium-derived microvesicles (annexin V+CD31+CD41–) in plasma of patients with advanced chronic kidney disease (*n* = 40), patients with transplantation (*n* = 40), and healthy subjects (*n* = 18) recruited from the University Hospital “12 de Octubre” (Madrid, Spain). Patients with kidney transplantation had B-cell lymphopenia, an impairment in co-stimulatory (CD86) and adhesion (CD54) molecules in monocytes, and a reduction in endothelium-derived microvesicles in plasma. The correlations between those parameters explained the modifications in the expression of co-stimulatory and adhesion molecules in monocytes caused by changes in lymphocyte populations, as well as the increase in the levels of endothelial-derived microvesicles in plasma caused by changes in lymphocyte and monocytes populations. Immunosuppressive treatment could directly or indirectly induce those changes. Nevertheless, the particular characteristics of these cells may partly explain the persistence of cardiovascular and renal alterations in patients who underwent transplantation, along with the decrease in arteriosclerotic events compared with advanced chronic kidney disease. In conclusion, the expression of adhesion molecules by monocytes and endothelial-derived microvesicles is related to lymphocyte alterations in patients with kidney transplantation.

## Introduction

Chronic kidney disease (CKD) is one of the leading causes of mortality and morbidity in developed countries (1). This pathology has a high frequency, affecting ~9% of the population worldwide (2). The incidence of CKD is expected to increase in the future (3) due to the increase in the prevalence of risk factors, such as hypertension and diabetes mellitus (2, 4, 5).

As CKD progresses and kidney function becomes less effective, various substances collectively termed uremic retention solutes accumulate in the body; those that exert adverse biological effects are termed uremic toxins. Uremic toxins are thought to contribute to inflammation, immune dysfunction, vascular disease, platelet dysfunction and increased bleeding risk, dysbiosis in the gut including increased translocation of bacteria, altered drug metabolism, as well as CKD progression (6–11). In addition, the risk of a cardiovascular event increases with decreasing renal glomerular filtration rate (GFR) and the occurrence of albuminuria (6, 12–14). The inflammatory state in advanced chronic kidney disease (ACKD) due to the inflammatory process in the kidney (15, 16) and the increase in excretion products in blood (e.g., uremic toxins or proinflammatory compounds) is particularly interesting (11, 17, 18). Both events lead to low-grade inflammation, similar to the basal inflammation observed in aging (4, 11, 17), which can be identified as inflamm-aging (19, 20). This low-grade inflammation is also associated with a worsening response to infections (21–23), an increased incidence of cancer (24, 25), and senescent phenotypes in immune cells and the vascular endothelium (4, 17, 18, 26–30). This inflammation and cellular senescence entail the development of associated pathologies, such as cardiovascular disease, which is the primary cause of mortality in CKD (4, 31). Proinflammatory monocytes (intermediate and non-classical) play a crucial role in the development of this pathology (4, 27, 30–33). In recent years, the extracellular vesicles—small particles which serve as a means of communication between cells—have captured the attention of researchers (34–37). The adhesion of monocytes to the vascular endothelium leads to release of proangiogenic factors and extracellular vesicles, including microvesicles (MV), by the endothelial cells, thereby inducing vascular damage (4, 27, 34, 38).

Most patients reaching end-stage kidney disease are treated with either dialysis or kidney transplantation (KT), which is currently the best available therapeutic option (39–41). However, KT does not entirely solve the problem primarily because the leading cause of CKD continues to affect the patient and prolongs the associated pathologies. Furthermore, other conditions, such as nephrotoxicity (42), anemia (43), oxidative stress (44), cardiovascular alterations (45, 46), or mineral-bone alterations (47) persist in patients who underwent transplantation. Moreover, immunosuppression (42), which is fundamental for avoiding transplant rejection, may modulate the low-grade basal inflammation. Currently, this potential relationship has not been extensively studied in situations of normal renal transplantation.

Numerous alterations associated with ACKD and its different treatments have been identified, including those that affect the immune and vascular systems. However, the approach through which these alterations can be corrected, at least partially by the KT procedure, is not well-established. Thus, the aim of the present study was to characterize the immune profile and MVs of patients with KT. This knowledge can be advantageous in designing strategies for monitoring patients and, above all, assessing the effectiveness of different treatments.

## Materials and Methods

### Study Population

**Figure 1:**
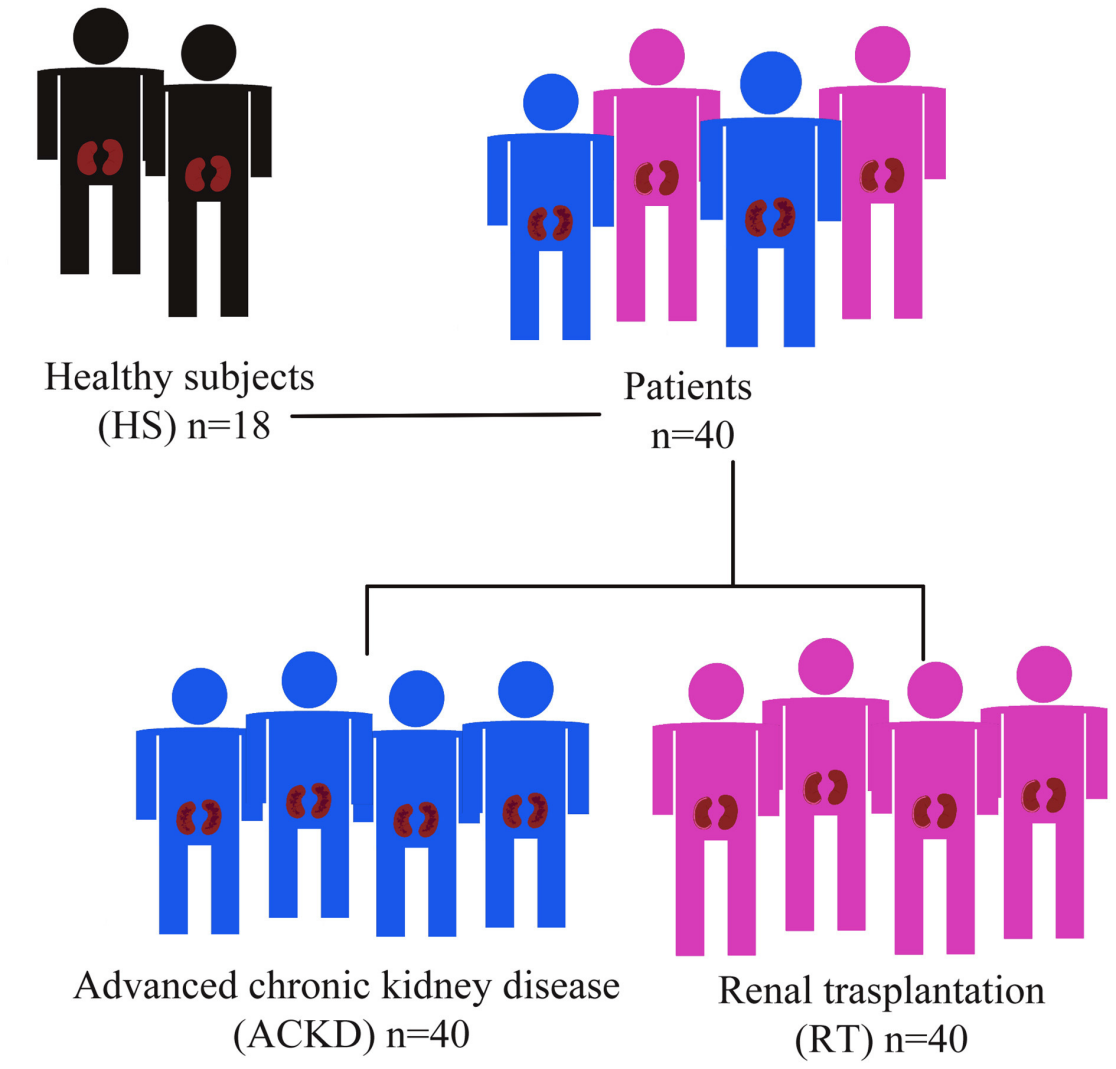
Description of cross-sectional study population.

We carried out a cross-sectional analysis involving 80 patients with CKD and 18 healthy subjects (HS) to establish standard criteria (Figure 1). Forty patients had stage 4–5 CKD, while the remaining 40 had received initial KT at least 6 months prior to sample collection. Patients with neoplasms, infections, and inflammatory or active autoimmune diseases were excluded. All patients were recruited at the Department of Nephrology, University Hospital “12 de Octubre” (Madrid, Spain). All procedures were performed according to the World Medical Association's Declaration of Helsinki and the protocol was approved by the Instituto de Investigación Sanitaria Hospital 12 de Octubre Ethics Committee (CEI: 17/407).

### Serum Sample Collection

Peripheral blood samples were obtained in ethylenediaminetetraacetic acid-coated tubes during routine medical reviews. All samples were analyzed within 18 h after collection. Biochemical and lymphocyte population characterizations were performed at the Department of Clinical Analysis and Department of Immunology of the “12 de Octubre” Hospital, respectively. Monocyte population and MV characterizations were conducted at the Department Genetics, Physiology, and Microbiology of Complutense University of Madrid (Spain). For MV characterization, plasma was obtained through centrifugation of blood samples at 1,500 × g for 20min. Plasma samples were stored at −20°C.

### Lymphocyte Characterization

Total lymphocytes, T lymphocytes (CD3+), T-helper lymphocytes (CD3+CD4+), T-cytotoxic lymphocytes (CD3+CD8+), B lymphocytes (CD3–CD19+), and natural killer (NK) cells (CD3–CD16+/CD56+) were analyzed (48, 49). Whole blood was stained using BD Multitest 6-color TBNK reagent (5:2 proportion; BD Biosciences, San José, CA, USA) for 15 min. Red blood cell lysis was performed using fluorescence-activated cell sorting (FACS) lysing solution (BD Biosciences). The lymphocyte subpopulations were determined using a FACSCanto II flow cytometer (BD Biosciences) and analyzed by the FACSCanto clinical software (BD Biosciences).

### Monocyte Characterization

Classical (CD14++CD16–), intermediate (CD14++CD16+) and non-classical (CD14+CD16+) monocyte populations were analyzed as previously described (50) with modifications. In addition, the expression of CD86/B-lymphocyte antigen B7-2 (CD86/B7-2) and CD54/intercellular adhesion molecule 1 (CD54/ICAM1) in each population was determined. A triple-staining immunofluorescence technique was utilized, and flow cytometry analysis was performed. Monoclonal antibodies conjugated with fluorochromes against CD14 (TuK4 clone, TRI-COLOR^®^ Invitrogen, Carlsbad, CA, USA), CD16 (3G8 clone, fluorescein isothiocyanate [FITC]; Invitrogen), CD86/B7.2 (BU63 clone, phycoerythrin; Biolegend, San Diego, CA, USA), and CD54/ICAM1 (MEM-111 clone, phycoerythrin; Invitrogen) were used.

**Figure 2:**
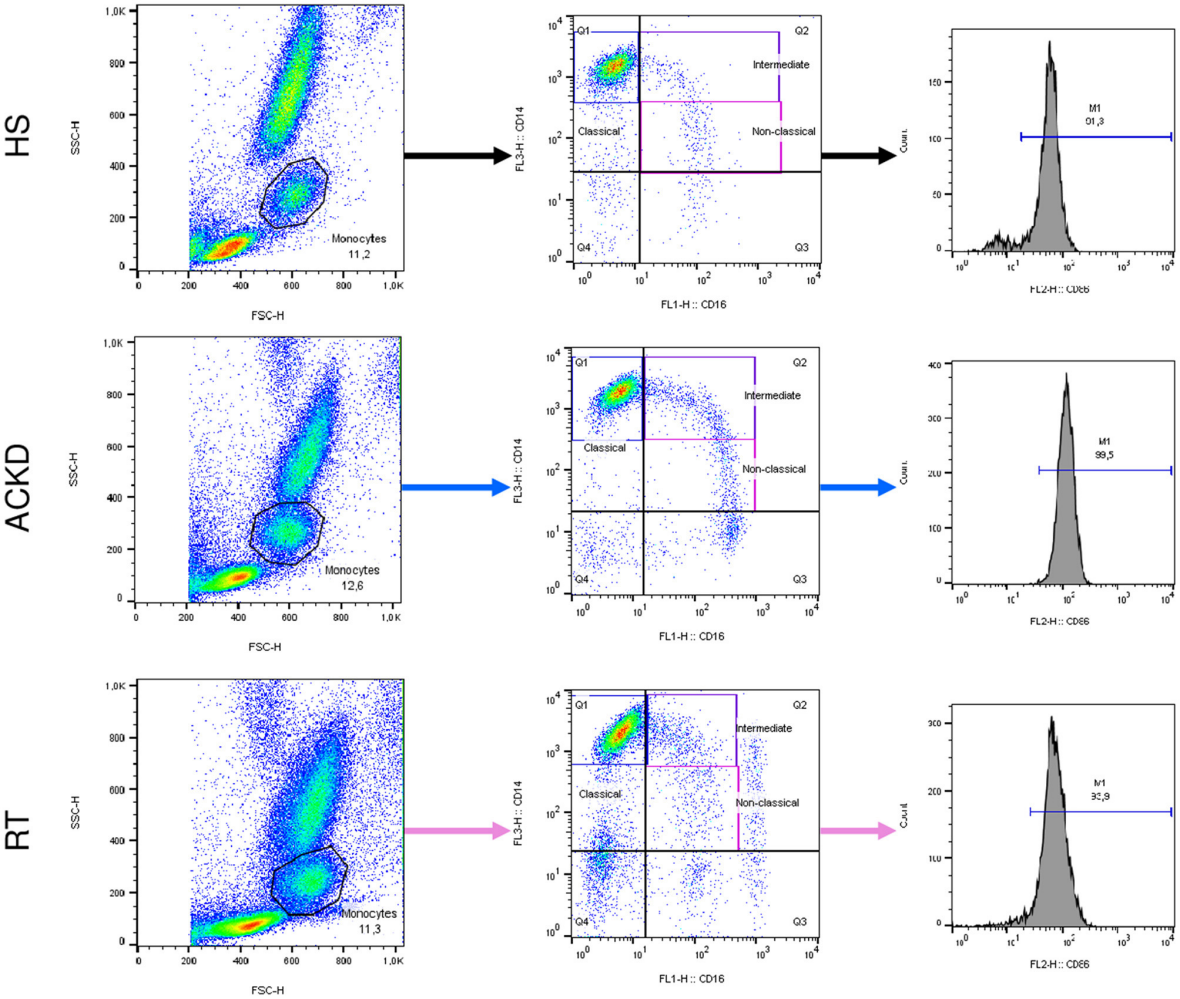
Representative flow cytometry findings of monocyte subsets and CD54/CD86 expression in the three groups: healthy subjects (HS), patients with advanced chronic kidney disease (ACKD), and patients with renal transplantation (RT). Monocyte subpopulations were assessed within the FSC-height/SSC-height. The classical (CD14++CD16–), intermediate (CD14++CD16+), and non-classical (CD14+CD16+) monocytes were evaluated using anti-CD16-FITC and anti-CD14–TRICOLOR. For each subpopulation, the expression of CD54 or CD86 was analyzed using anti-CD54-PE or anti-CD86-PE. FITC, fluorescein isothiocyanate; FSC, forward scatter; PE, phycoerythrin; SSC, side scatter.

Briefly, whole blood was incubated with the corresponding antibody for 25 min at room temperature in darkness. Red blood lysis was performed using FACS Lysing Solution (BD Biosciences) for 10 min prior to centrifugation at 400 × g. The cells were fixed using Cell Fix (BD Biosciences) and stored at 4°C until assessment. The maximum storage period was 24 h. The monocyte subpopulations and phenotypes were determined using a FACSCalibur cytometer (BD Biosciences), with the support of the staff of the cytometry associated research center of Complutense University of Madrid (Spain) and analyzed by the FlowJo™ software (Ashland, OR, USA). The results were expressed as the percentage of monocyte subtype with respect to the total monocyte population, in the case of three subtypes of monocytes. Alternatively, data were presented as the percentage of each population that expressed CD86/CD54 and the mean fluorescence intensity (MFI), which represents the amount of molecule expressed by each monocyte (Figure 2).

### MV Characterization

The total number of MVs (AnnexinV+) and endothelial-derived MVs (AnnexinV+CD31+CD41–), as well as the expression of tissue factor (CD142) in endothelial-derived MVs, were determined as previously described (49). A quadruple-staning immunofluorescence technique was utilized, and flow cytometry analysis was performed. Monoclonal antibodies conjugated with fluorochromes against Annexin V (Annexin V-FITC Kit; Miltenyi Biotec, Bergisch Gladbach, Germany), CD41/integrin subunit alpha 2b (MEM-06 clone, peridnine chlorophyll protein; Invitrogen), CD31/platelet and endothelial cell adhesion molecule 1 [PECAM1] (WM-59 clone, phycoerythrin; BD Bioscience), and CD142/tissue factor (HTF-1 clone, allophycocyanin [APC]; Invitrogen) were used.

Briefly, platelet-free plasma samples were centrifuged at 110,000 × g for 2 min and resuspended in Annexin-V binding buffer (Annexin V-FITC Kit; Miltenyi Biotec). Subsequently, the samples were incubated with the corresponding antibodies for 40 min at room temperature in darkness, fixed using Cell Fix (BD Bioscience), and stored at 4°C until assessment. The maximum storage period was 24 h. The MV subpopulations were characterized through flow cytometry using a FACSCalibur cytometer (BD Biosciences) with the support of the staff of the cytometry associated research center of Complutense University of Madrid (Spain) and analyzed by the FlowJo™ software. The standardization on the FACSCalibur device was carried out as previously described (49).

### Statistical Analysis

SPSS version 21.0 (Armonk, NY, USA) was used for the statistical analysis. The data were expressed as the mean ± standard deviation. The normality of the samples and variance homogeneity were checked using one-sample Kolmogorov–Smirnov and Levene tests. Normal variables were evaluated using one-way analysis of variance to determine individual differences for each parameter followed by *post-hoc* analysis. The *post-hoc* analysis was performed using Tukey's test for variables with homogeneous variances and the Games–Howell test for those with heterogeneous variances. For non-normal variables, the Mann–Whitney *U*-test was performed. For qualitative data, the chi-squared test was performed, and the results were expressed as relative and absolute frequencies. The Spearman correlation test was carried out for correlation analysis between lymphocytes, monocytes, and MV subpopulations in renal transplant patients. *P* ≤ 0.05 denoted statistical significance.

## Results

### Population Description

The baseline characteristics of patients with CKD and HS are shown in Table 1. There was no difference between the age or sex of patients with ACKD (61 ± 17 years; 65% males) and transplantation (54 ± 12 years; 68% males). The numbers of individuals affected by hypertension and diabetes mellitus were similar in both groups of patients (ACKD: 90% and 45%; KT: 98% and 40%, respectively) as well as the smoking habit (ACKD: 28%; KT: 25%). Whereas de number of patients with dyslipidemia and hyperuricemia was higher in ACKD (78%, *p* = 0.034; 70%, *p* = 0.002, respectively) than in kidney transplantation (53; 33%, respectively). The estimated GFR was lower in patients with ACKD (16 ± 17 mL/min/1.73 m^2^, *p* = 0.000) than in those with kidney transplantation (49 ± 19 mL/min/1.73 m^2^). Moreover, there were no differences in the levels of C-reactive protein in both patient groups (ACKD: 0.45 ± 0.44 mg/dL; KT: 0.47 ± 0.89 mg/dL).

**Table 1:** Baseline characteristics of patients and healthy subjects.

	**HS**	**Patients with ACKD**	**Patients with KT**
***n***	18	40	40
**Age (years), mean ± SD^b^**	51 ± 16	61 ± 17	54 ± 12
**Male**, ***n*** **(%)^a^**	9 (50%)	26 (65%)	27 (68%)
**Etiopathology**, ***n*** **(%)^a^**			
Nephroangiosclerosis	–	7 (17.5%)	6 (15%)
Diabetic nephropathy	–	13 (32.5%)	8 (20%)
Glomerular nephropathy	–	6 (15%)	4 (10%)
Polycystic kidney disease	–	4 (10%)	8 (20%)
Interstitial nephritis	–	6 (15%)	2 (5%)
Others	–	4 (10%)	10 (25%)
**Hypertension**, ***n*** **(%)^a^**	1 (6%)	36 (90%)^***^	39 (98%)^***^
**Diabetes mellitus**, ***n*** **(%)^a^**	2 (11%)	18 (45%)^*^	16 (40%)^*^
**Dyslipidemia**, ***n*** **(%)^a^**	0 (0%)	31 (78%)^***^	21 (53%)^***^^#^
**Hyperuricemia**, ***n*** **(%)^a^**	0 (0%)	28 (70%)^***^	13 (33%)^***^^##^
**Smoking**, ***n*** **(%)^a^**	4 (22%)	11 (28%)	10 (25%)
**eGFR (mL/min/1.73 m** ^**2**^ **), mean ± SD^d^**	>90	16 ± 17^***^	49 ± 19^***^^###^
**Serum creatinine (mg/dL), mean ± SD^c^**	0.8 ± 0.2	4.2 ± 1.0^***^	1.5 ± 0.5^***^^###^
**Serum albumin (mg/dL), mean ± SD^b^**	4.7 ± 0.3	4.3 ± 0.4^***^	4.5 ± 0.4^###^
**Proteins (mg/dL), mean ± SD^b^**	7.1 ± 0.4	6.9 ± 0.5	7.0 ± 0.6
**CRP (mg/dL), mean ± SD^d^**	0.27 ± 0.5	0.45 ± 0.44^**^	0.47 ± 0.89^*^

a *Chi-squared test*.

b *ANOVA (Tukey test)*.

c ANOVA (Games–Howell test).

d *Mann–Whitney U-test. Statistical significance was denoted by*

* *p ≤ 0.05*,

** *p ≤ 0.01*,

*** *p ≤ 0.001 vs. HS*;

# *p ≤ 0.05*,

## *p ≤ 0.01*,

### *p ≤ 0.001 vs. ACKD*.

*ACKD, advanced chronic kidney disease; ANOVA, analysis of variance; CRP, C-reactive protein; eGFR, estimated glomerular filtration rate; HS, healthy subjects; KT, kidney transplantation; SD, standard deviation*.

Regarding the immunosuppressive treatment, the most used treatment was a combination of tacrolimus and mycophenolic acid (26 patients, 65%), followed by a combination of tacrolimus and everolimus (seven patients, 17.5%).

### Lymphocyte Characterization

**Figure 3:**
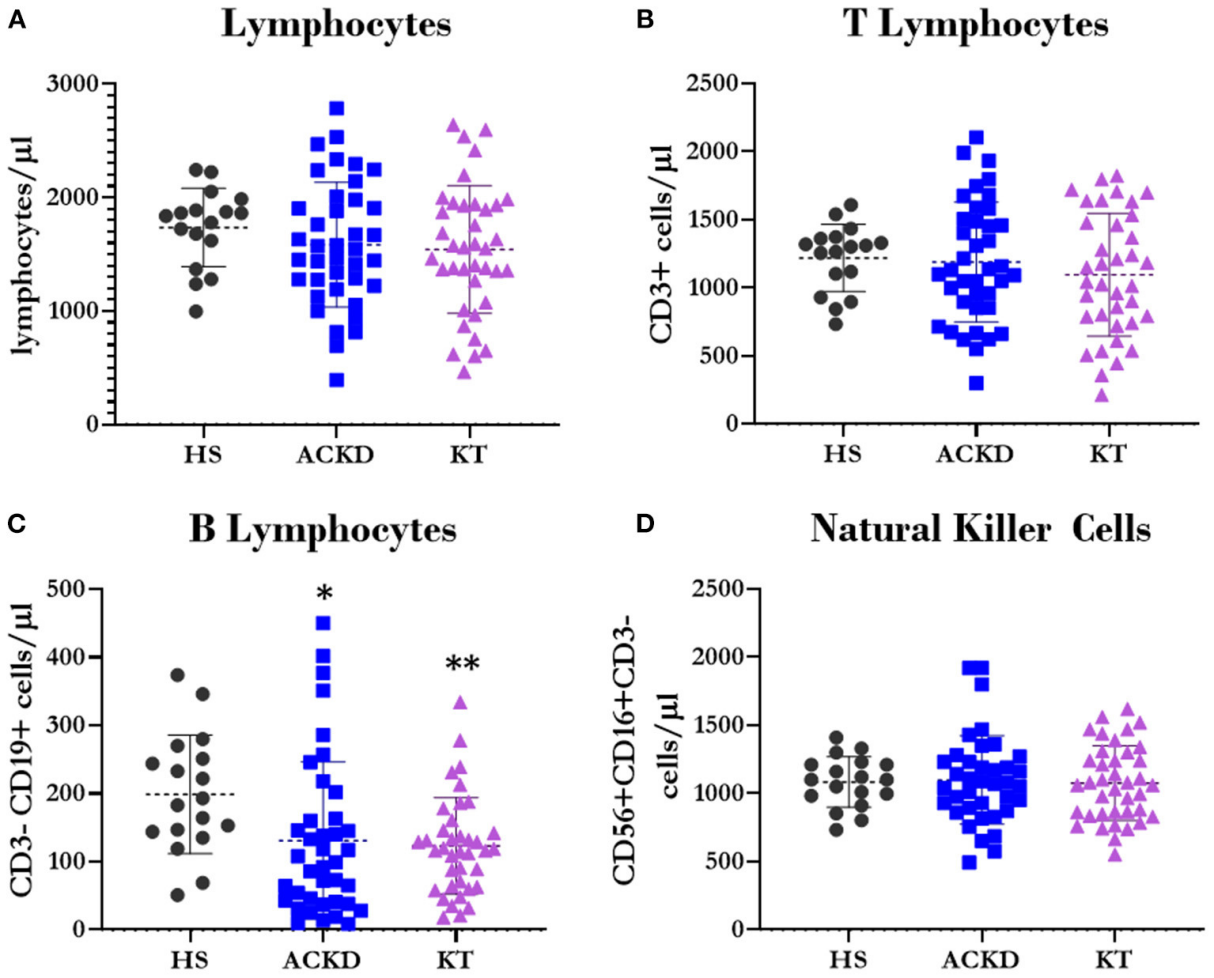
Description of lymphocyte subpopulations. Number of total lymphocytes^*Ψ*^ **(A)**, T lymphocytes^*Ω*^ **(B)** (CD3+), B lymphocytes^*Ω*^ **(C)** (CD3–CD19+), and natural killer cells^*Ψ*^ **(D)** (CD56+CD16+CD3–) in healthy subjects (HS), patients with advanced chronic kidney disease (ACKD), and patients with kidney transplantation (KT). ^*^p ≤ 0.05, ^**^p ≤ 0.01 vs. HS. Statistical analysis: ^*Ψ*^ANOVA (Tukey test). ^*Ω*^ANOVA (Games–Howell test). ANOVA, analysis of variance.

The present results did not show any differences in the total number of lymphocytes (Figure 3A), T lymphocytes (Figure 3B), or NK cells (Figure 3D). A decrease in the number of B lymphocytes was observed in ACKD (130.86 ± 155.55 cells/μL) and KT (123.05 ± 71.07 cells/μL) patients vs. HS (198.77 ± 87.08 cells/μL, *p* = 0.047 and 0.009, respectively) (Figure 3C).

**Figure 4:**
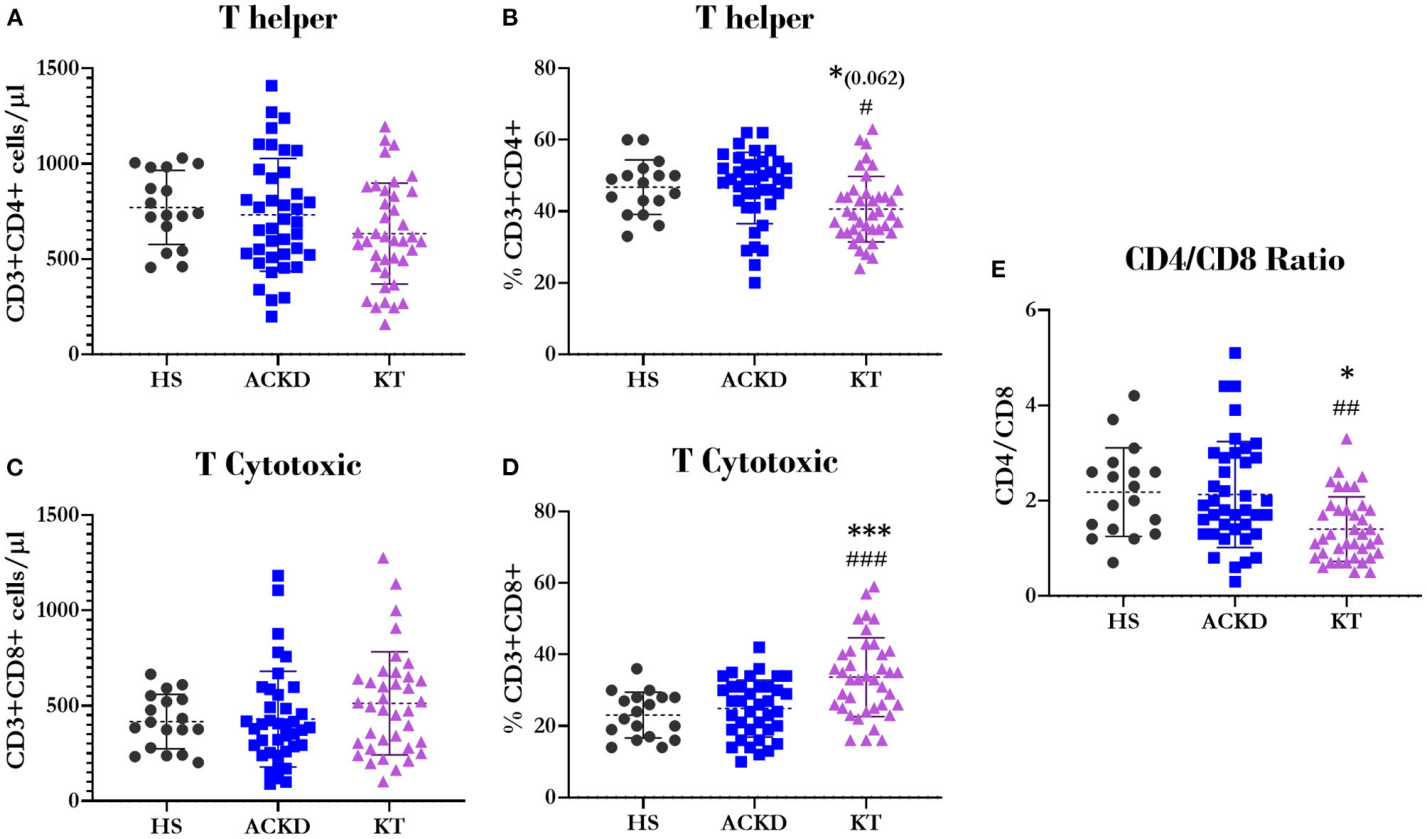
Description of T lymphocyte subpopulations. Number^*Ψ*^ **(A)** and percentage^*Ψ*^ **(B)** of T-helper lymphocytes (CD3+CD4+); number^*Ψ*^ **(C)** and percentage^*Ψ*^ **(D)** of T-cytotoxic lymphocytes (CD3+CD8+); relationship between helper and cytotoxic lymphocytes^*Ω*^ **(E)** (CD4/CD8 ratio) in healthy subjects (HS), patients with advanced chronic kidney disease (ACKD), and patients with kidney transplantation (KT). ^*^p ≤ 0.05, ^***^p ≤ 0.001 vs. HS; ^#^*p* ≤ 0.05, ^##^*p* ≤ 0.01, and ^###^*p* ≤ 0.001 vs. ACKD. Statistical analysis: ^*Ψ*^ANOVA (Tukey test). ^*Ω*^ANOVA (Games–Howell test). ANOVA, analysis of variance.

Regarding the T-lymphocyte subpopulations, we did not find differences in the total numbers of T-helper and T-cytotoxic cells (Figures 4A,C, respectively). Nevertheless, patients with KT showed a decrease in the percentage of T-helper lymphocytes (40.62 ± 9.16%, *p* = 0.062 vs. HS and *p* = 0.016 vs. ACKD; Figure 4B). They also exhibited an increase in the cytotoxic subpopulation (33.67 ± 11.01%, *p* = 0.000; Figure 4D) compared with HS (46.76 ± 7.64 and 23.06 ± 6.41%, respectively) and patients with ACKD (46.51 ± 9.96 and 24.89 ± 7.97%, respectively), resulting in a decreased CD4/CD8 ratio (KT: 1.4 ± 0.68, HS: 2.18 ± 0.93, *p* = 0.011; ACKD: 2.13 ± 1.11, *p* = 0.002; Figure 4E).

### Monocyte Characterization

**Figure 5:**
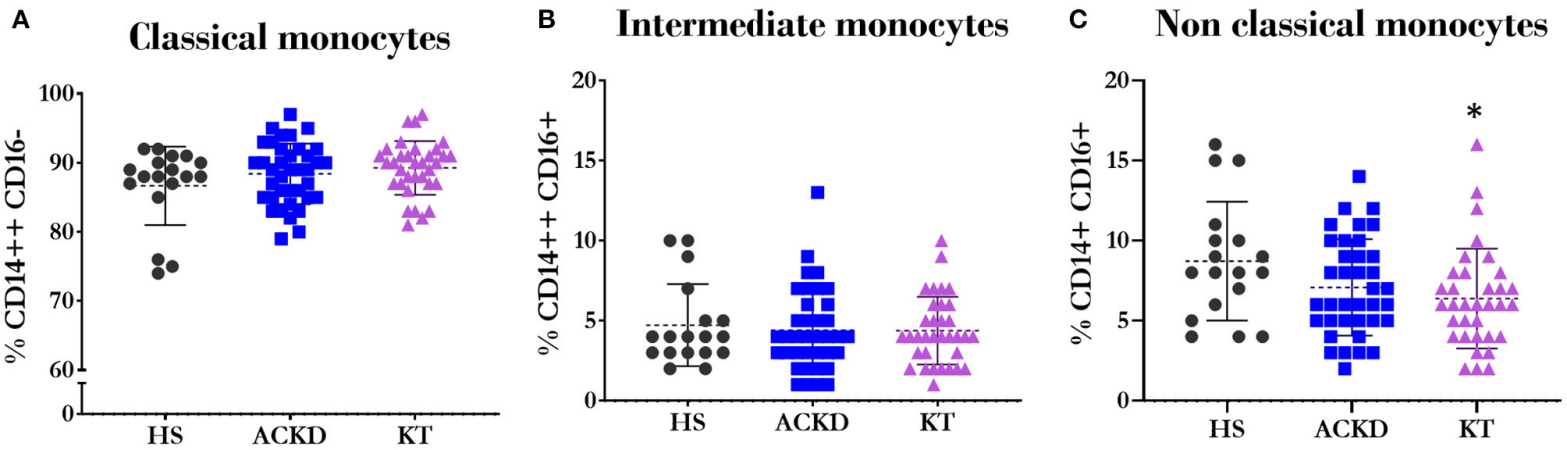
Description of monocyte subsets. Percentage of classical^*Φ*^ **(A)** (CD14++CD16−); intermediate^*Ψ*^ **(B)** (CD14++CD16+) and non-classical^*Ψ*^ **(C)** (CD14+CD16+) monocytes in healthy subjects (HS), patients with advanced chronic kidney disease (ACKD), and patients with kidney transplantation (KT). ^*^p ≤ 0.05 vs. HS. Statistical analysis: ^*Ψ*^ANOVA (Tukey test). ^*Ψ*^Mann–Whitney *U*-test. ANOVA, analysis of variance.

There were no differences between groups in the percentages of classical (Figure 5A) and intermediate (Figure 5B) monocytes. Patients with KT had a lower percentage of non-classical monocytes (6.38 ± 3.11%) (Figure 5C) compared with HS (8.72 ± 3.7%, *p* = 0.036).

**Figure 6:**
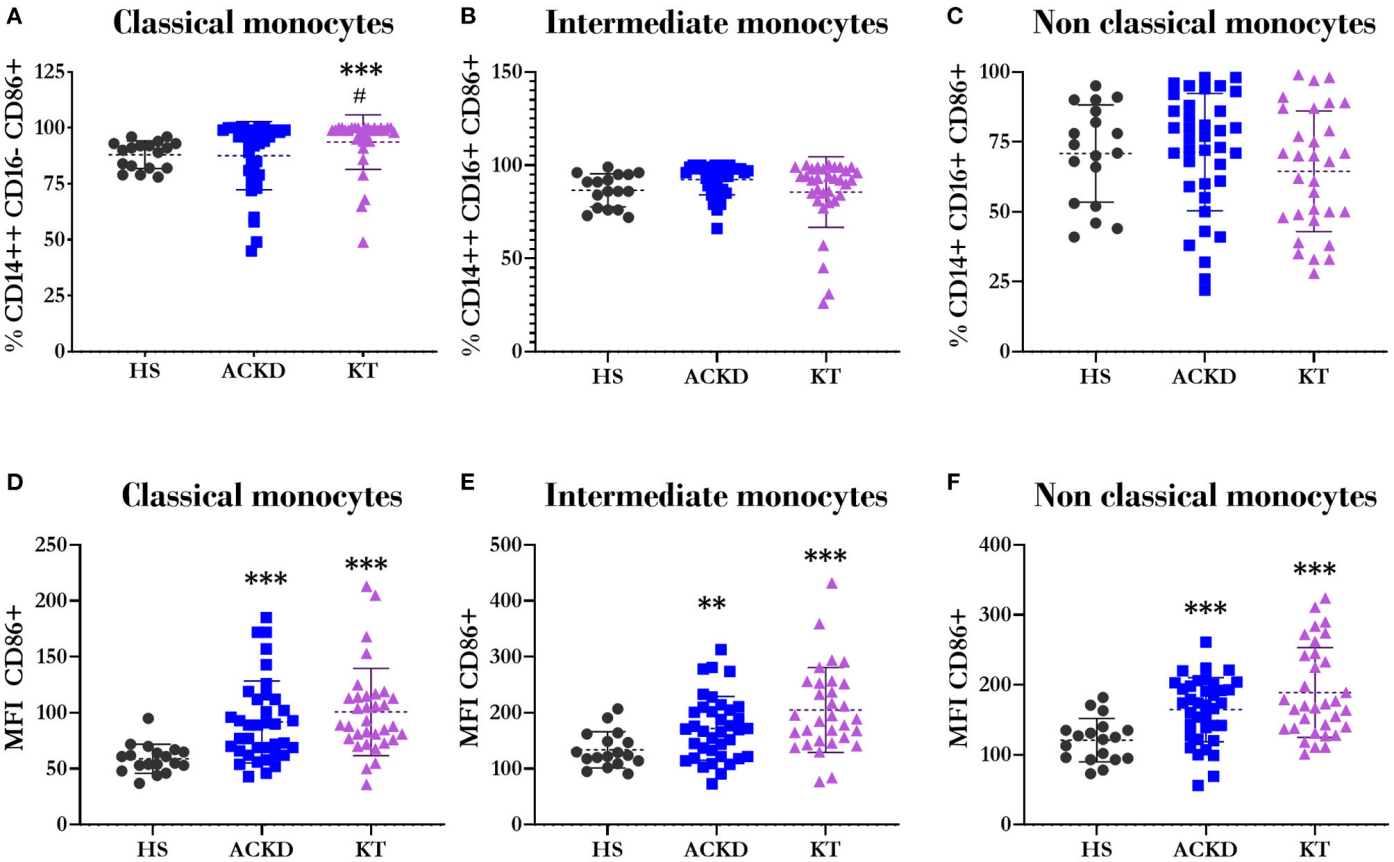
Expression of CD86/B7.2 in different monocyte subsets. Percentage of classical^Φ^ **(A)** (CD14++CD16–), intermediate^*Ω*^ **(B)** (CD14++CD16+), and non-classical^*Ψ*^ **(C)** (CD14+CD16+) monocytes expressing CD86. Mean fluorescence intensity of CD86 in classical^*Ω*^ **(D)** (CD14++CD16–), intermediate^*Ω*^ **(E)** (CD14++CD16+), and non-classical^*Ω*^ **(F)** (CD14+CD16+) monocytes in healthy subjects (HS), patients with advanced chronic kidney disease (ACKD), and patients with kidney transplantation (KT). ^**^p ≤ 0.01, ^***^p ≤ 0.001 vs. HS; ^#^*p* ≤ 0.05 vs. ACKD. Statistical analysis: ^*Ψ*^ANOVA (Tukey test). ^*Ω*^ANOVA (Games–Howell test). ^Φ^Mann–Whitney *U-*test. ANOVA, analysis of variance.

However, the most notable differences were observed in CD86 and CD54 in different subpopulations. There was a higher percentage of classical monocytes that express CD86 in KT patients (93.63 ± 11.99%) vs. HS (88 ± 6.15%, *p* = 0.000) and ACKD patients (87.92 ± 15.15%, *p* = 0.03; Figure 6A). Notably, the percentage of monocytes expressing this molecule did not change in the intermediate (Figure 6B) and non-classical (Figure 6C) subtypes. Meanwhile, the number of cells expressing CD86 was increased in patients with ACKD (classical: 91.76 ± 36.68 MFI, *p* = 0.000; intermediate: 172.21 ± 56.94 MFI, *p* = 0.008; non-classical: 164.43 ± 45.79 MFI, *p* = 0.000) and those with KT (classical: 100.7 ± 38.97 MFI, *p* = 0.000; intermediate: 208.88 ± 78.01 MFI, *p* = 0.000; non-classical: 188.97 ± 64.18 MFI, *p* = 0.000) compared with HS (classical: 58.94 ± 12.98 MFI; intermediate: 133.76 ± 32.41 MFI; non-classical: 120.78 ± 31 MFI) in the three monocyte subpopulations (Figures 6D–F).

**Figure 7:**
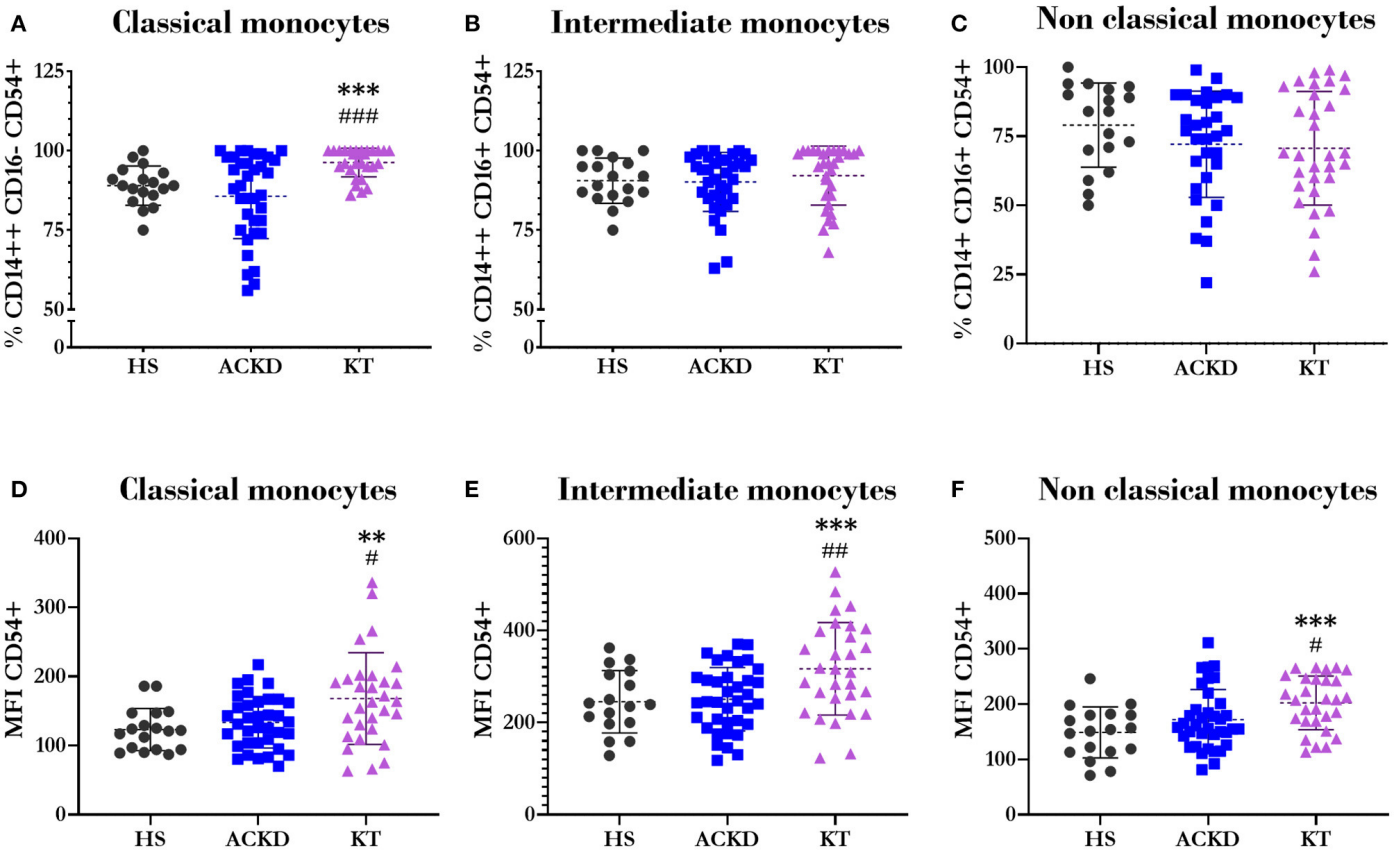
Expression of CD54/ICAM1 in different monocyte subsets. Percentage of classical^*Ω*^ **(A)** (CD14++CD16−), intermediate^*Ψ*^ **(B)** (CD14++CD16+), and non-classical^*Ψ*^ **(C)** (CD14+CD16+) monocytes expressing CD54. Mean fluorescence intensity of CD54 in classical^*Ω*^ **(D)** (CD14++CD16−), intermediate^*Ψ*^ **(E)** (CD14++CD16+), and non-classical^*Ψ*^ **(F)** (CD14+CD16+) monocytes in healthy subjects (HS), patients with advanced chronic kidney disease (ACKD), and patients with kidney transplantation (KT). ^**^p ≤ 0.01, ^***^p ≤ 0.001 vs. HS; ^#^*p* ≤ 0.05, ^##^*p* ≤ 0.01, ^###^*p* ≤ 0.001 vs. ACKD. Statistical analysis: ^*Ψ*^ANOVA (Tukey test). ^*Ω*^ANOVA (Games-Howell test). ANOVA, analysis of variance.

Regarding the expression of CD54 in the different subsets of monocytes, there was an increase in the percentage of classical monocytes expressing this molecule in patients with KT (96.13 ± 4.49%) vs. HS (89 ± 6.18%, *p* = 0.001) and patients with ACKD (85.95 ± 13.25%, *p* = 0.000; Figure 7A). However, there was no difference in the percentage of intermediate (Figure 7B) and non-classical monocytes (Figure 7C). The expression level of CD54 in the three subsets (Figures 7D–F) was higher in patients with KT (classical: 167.9 ± 66.43 MFI; intermediate: 316.6 ± 100.41 MFI; non-classical: 210 ± 64.06 MFI) compared with HS (classical: 123.11 ± 30.55 MFI, *p* = 0.008; intermediate: 245.18 ± 67.73, *p* = 0.014; non-classical: 148.78 ± 46.05 MFI, *p* = 0.001), and patients with ACKD (classical: 133.67 ± 37.29 MFI, *p* = 0.041; intermediate: 250.51 ± 68.92 MFI, *p* = 0.004; non-classical: 171.92 ± 54.24 MFI, *p* = 0.02).

### MVs Characterization

**Figure 8:**
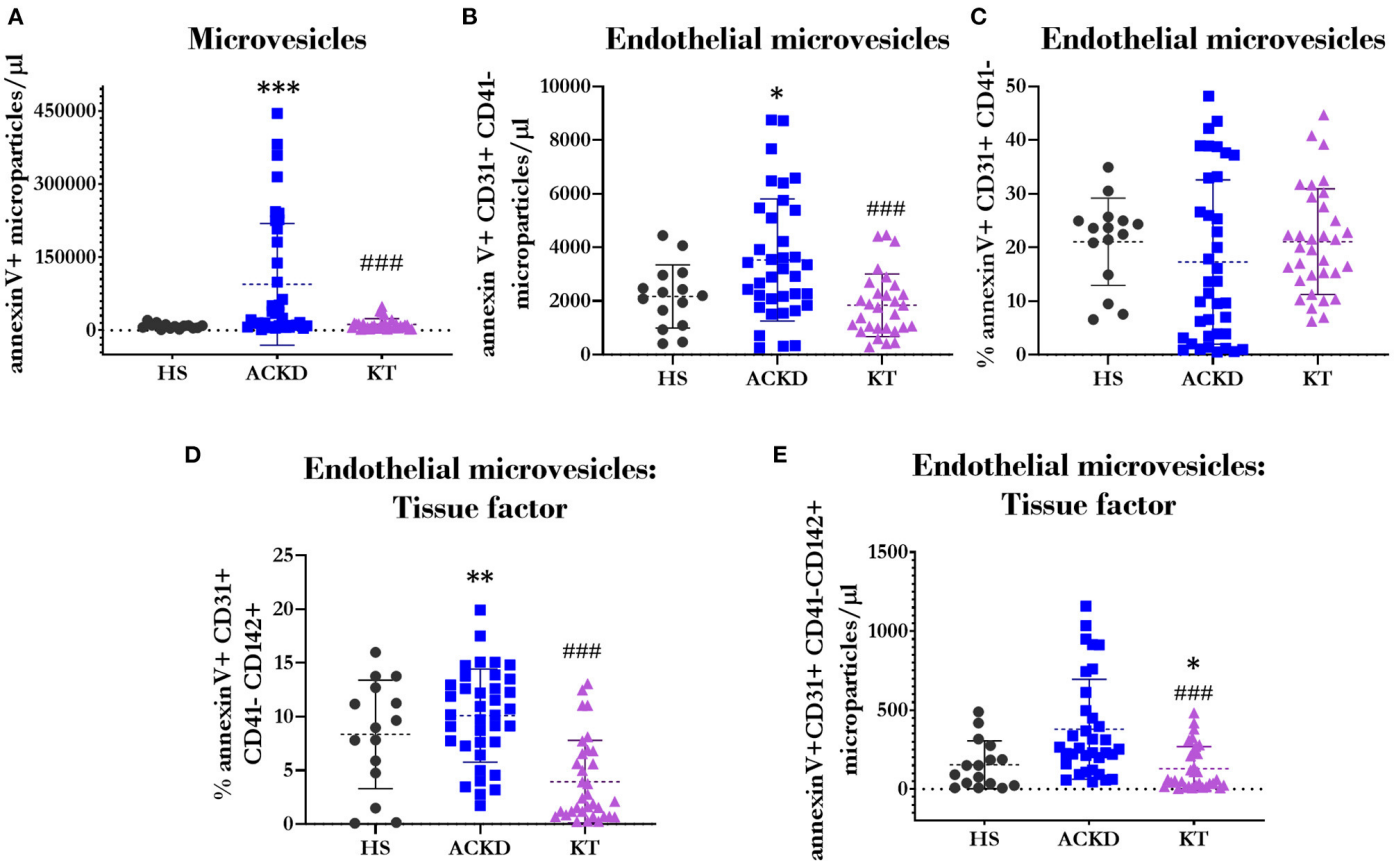
Description of the microvesicles phenotype. Total number of microvesicles^Φ^ **(A)** (annexin V+); number^*Ω*^ **(B)** and percentage^*Ω*^ **(C)** of endothelial microvesicles (annexin V+CD31+CD41–); and percentage^*Ψ*^ **(D)** and number^Φ^ **(E)** of endothelial microvesicles expressing tissue factor (CD142) in healthy subjects (HS), patients with advanced chronic kidney disease (ACKD), and patients with kidney transplantation (KT). ^*^p ≤ 0.05, ^**^p ≤ 0.01, and ^***^p ≤ 0.001 vs. HS; ^###^*p* ≤ 0.001 vs. ACKD. Statistical analysis: ^*Ψ*^ANOVA (Tukey test). ^*Ω*^ANOVA (Games–Howell test). ^Φ^Mann–Whitney *U-*test. ANOVA, analysis of variance.

The total numbers of MVs (Figure 8A) and endothelial MVs (Figure 8B) were increased in patients with ACKD (94,335.97 ± 124,672 MVs/μL; 66,355.47 ± 124,672.09 MVs/μL; respectively) compared with HS (8,599.14 ± 5,341.19 MVs/μL, *p* = 0.001; 7,417.7 ± 11,418.63 MVs/μL, *p* = 0.02; respectively) and patients with KT (12,286.87 ± 11,637.93 MVs/μL, *p* = 0.001; 6,412.73 ± 764.68 MVs/μL, *p* = 0.001; respectively). There were no differences observed in the percentage of endothelial MVs (Figure 8C). The percentage of endothelial MVs expressing tissue factor (CD142) (Figure 8D) was higher in patients with ACKD (8,327.29 ± 1,736.99%) vs. HS (664.29 ± 703.9%, *p* = 0.003) and patients with KT (845.76 ± 1,390%, *p* = 0.000). A lower number of endothelial MVs expressing tissue factor (Figure 8E) was observed in patients with KT (128.78 ± 139.2 MVs/μL) compared with HS (153.5 ± 151.55 MVs/μL, *p* = 0.017) and patients with ACKD (378.68 ± 315.89 MVs/μL, *p* = 0.000).

### Correlations

**Figure 9:**
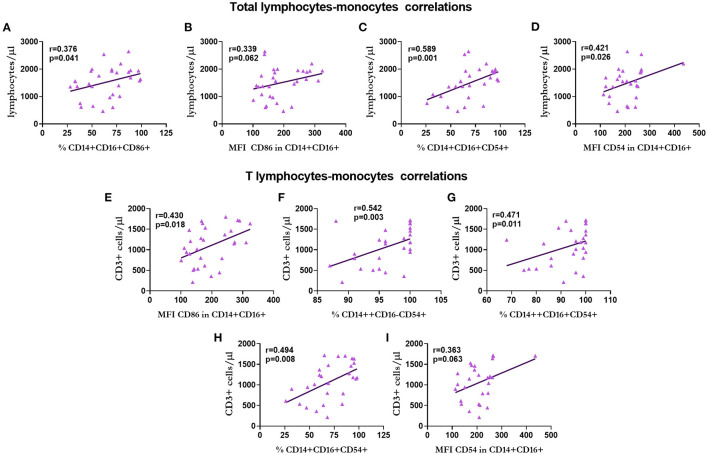
Correlation between the number of total lymphocytes and T lymphocytes with monocytes in renal transplantation. Correlation of the total number of lymphocytes with the percentage of non-classical monocytes CD86+ **(A)**, expression of CD86 in non-classical monocytes **(B)**, percentage of non-classical monocytes expressing CD54 **(C)**, and expression of CD54 in non-classical monocytes **(D)**. Correlations of T lymphocytes with the expression of CD86 in non-classical monocytes **(E)**, percentage of classical **(F)**, intermediate **(G)**, and non-classical **(H)** CD54+ monocytes, and the expression of CD54 in non-classical monocytes **(I)** are shown.

**Figure 10:**
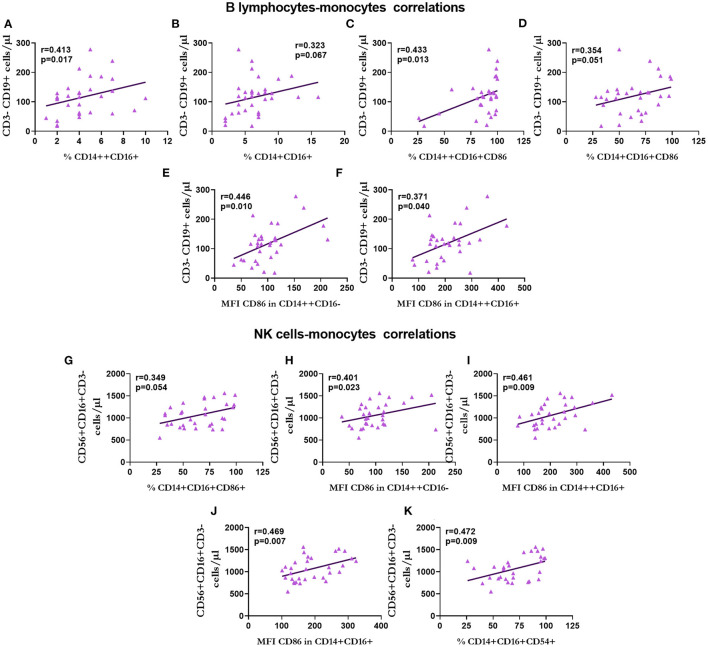
Correlation between the number of B lymphocytes and natural killer (NK) cells with monocytes in renal transplantation. Correlation between B lymphocytes and the percentage of intermediate **(A)** and non-classical **(B)** monocytes, the percentage of intermediate **(C)** and non-classical **(D)** CD86+ monocytes and the expression of CD86 in classical **(E)** and intermediate **(F)** monocytes. Correlations of NK cells with the percentage of CD86+ monocytes **(G)**, expression of CD86 in classical **(H)**, intermediate **(I)**, and non-classical **(J)** monocytes, and the percentage of non-classical monocytes expressing CD54 **(K)** are shown.

**Figure 11:**
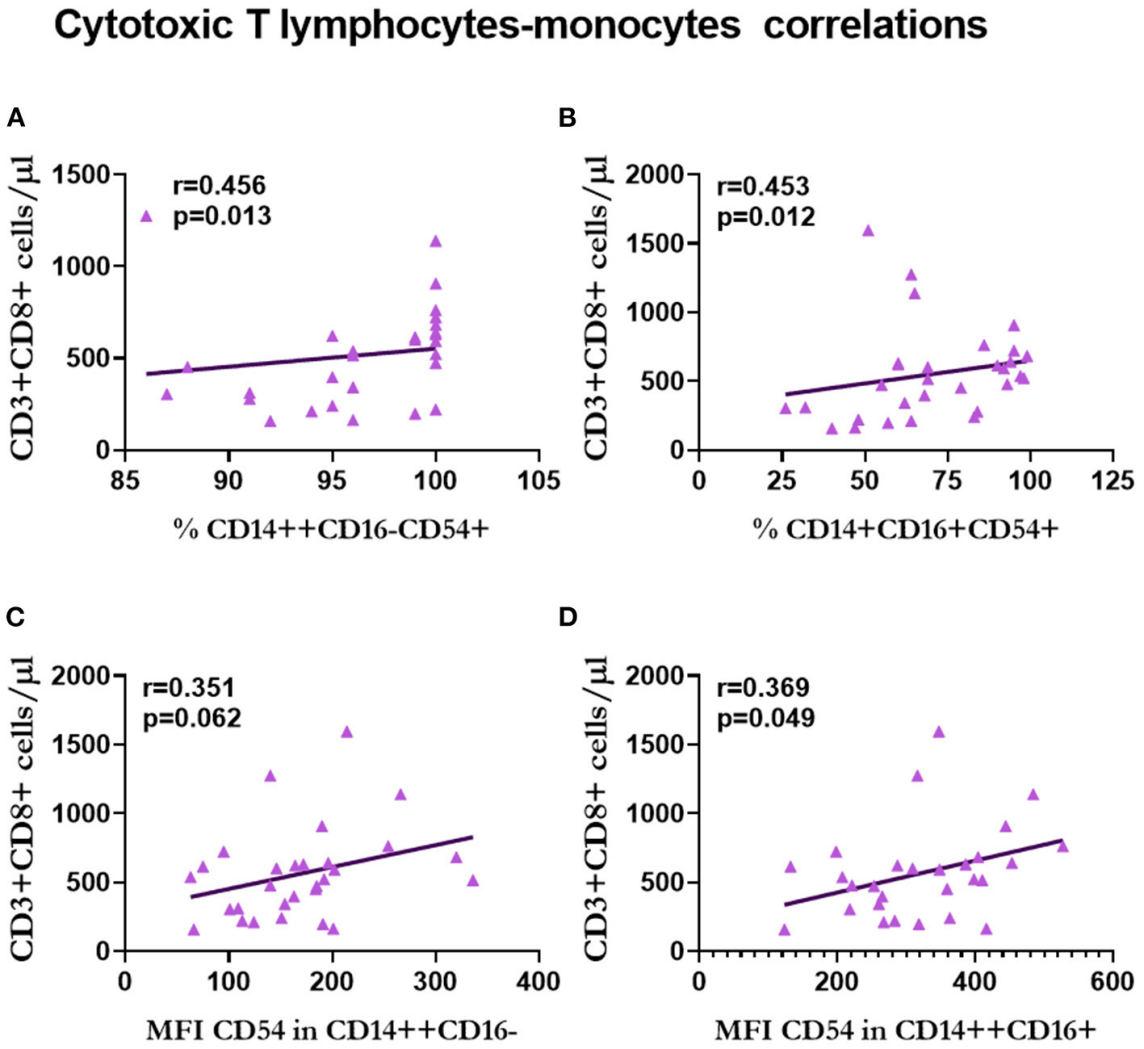
Correlation between T-cytotoxic lymphocytes and monocytes in renal transplantation. Correlations between T-cytotoxic lymphocytes and the percentage of classical **(A)** and non-classical **(B)** CD54+ monocytes, and the expression of CD54 in classical **(C)** and intermediate **(D)** monocytes are shown.

**Figure 12:**
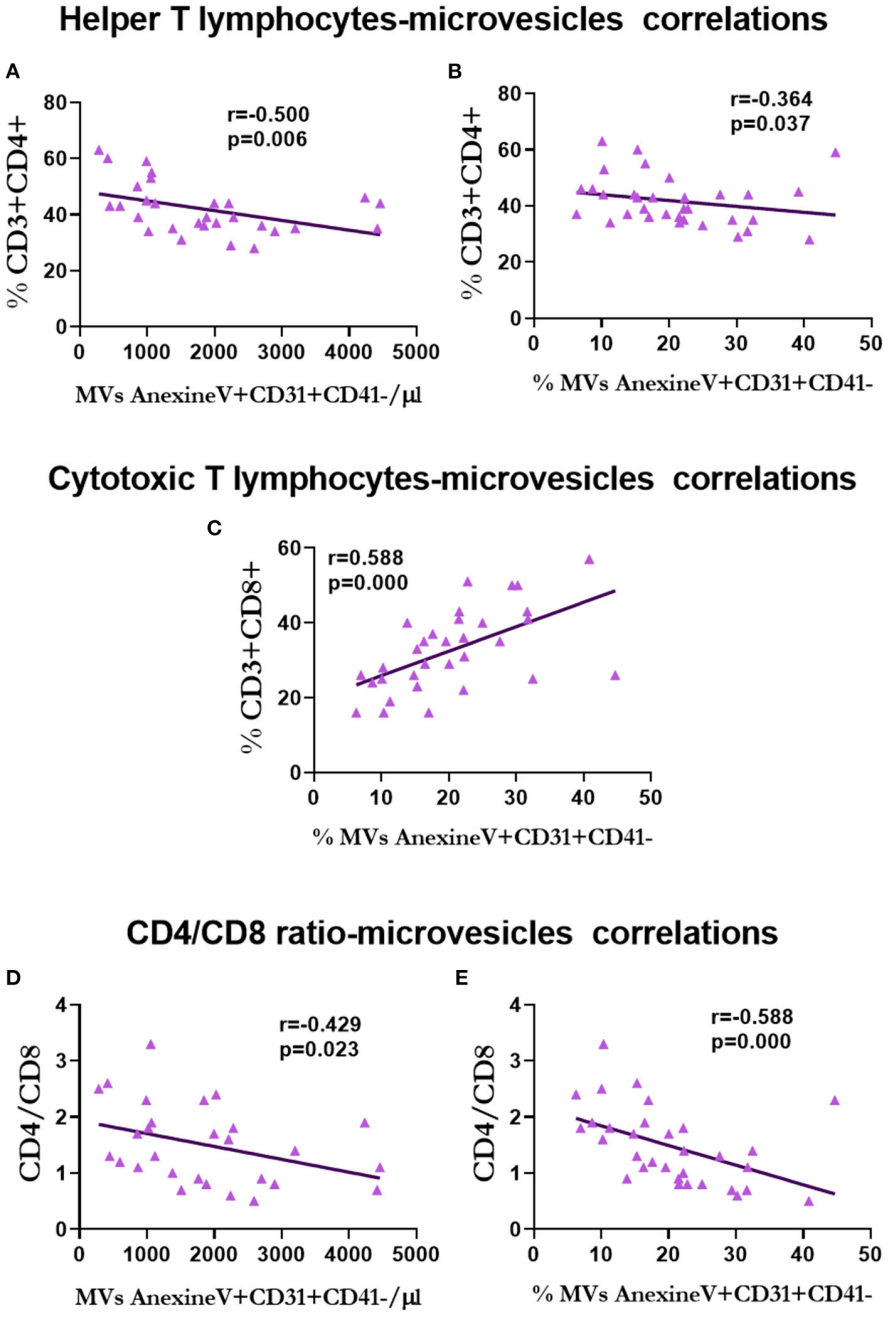
Correlation between lymphocytes and microvesicles in renal transplantation. Correlation between T-helper lymphocytes and the number **(A)** and percentage **(B)** of endothelium microvesicles. Correlation between T-cytotoxic lymphocytes and percentage of endothelium microvesicles **(C)**. Correlation of CD4/CD8 ratio with the number **(D)** and percentage **(E)** of endothelium microvesicles.

**Figure 13:**
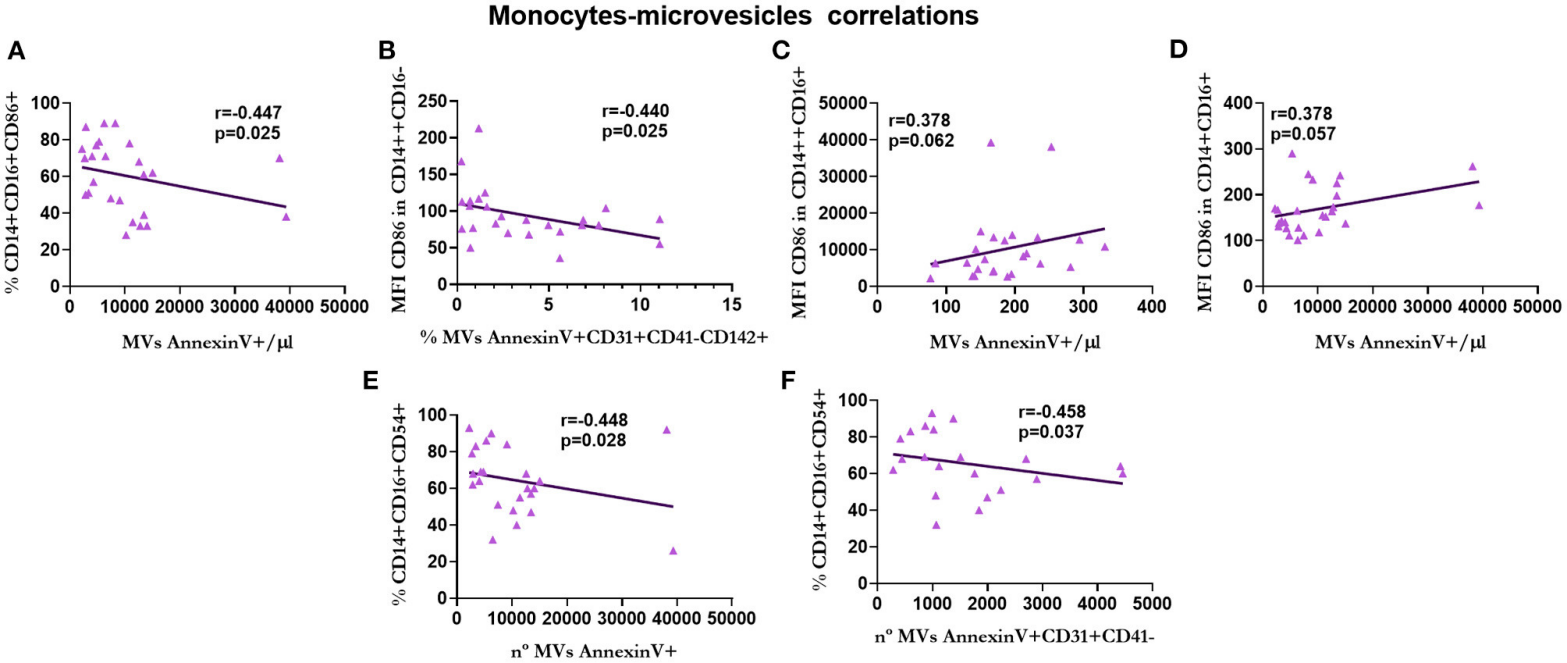
Correlation between monocytes and microvesicles in renal transplantation. Correlations between the percentage of CD86+ non-classical monocytes and the total number of microvesicles **(A)**, the expression of CD86 in classical monocytes and the percentage of 142+ endothelium microvesicles **(B)**, the expression of CD86 in intermediate monocytes and the total number of microvesicles **(C)**, the expression of CD86 in non-classical monocytes and the total number of microvesicles **(D)**, the percentage of CD54+ intermediate monocytes and the total number of microvesicles **(E)**, and the percentage of CD54+ intermediate monocytes and the number of endothelium microvesicles **(F)** are shown.

The correlations between the subpopulations of lymphocytes, monocytes, and MVs in patients with KT are shown in Figures 9–**13**.

The total number of lymphocytes showed a positive correlation with the percentage (*r* = 0.376, *p* = 0.041; Figure 9A) and expression (*r* = 0.339, *p* = 0.062 statistical trend; Figure 9B) of CD86 and percentage (*r* = 0.589, *p* = 0.001; Figure 9C) and expression (*r* = 0.421, *p* = 0.026; Figure 9D) of CD54 in non-classical monocytes in all cases.

Regarding T lymphocytes, we observed a positive correlation with the expression of CD86 in non-classical monocytes (*r* = 0.430, *p* = 0.018; Figure 9E), the percentage of classical (*r* = 0.430, *p* = 0.018; Figure 9F), intermediate (*r* = 0.471, *p* = 0.011; Figure 9G) and non-classical (*r* = 0.494, *p* = 0.008, Figure 9H) CD54+ monocytes, and the expression of CD54 in non-classical monocytes (*r* = 0.363, *p* = 0.063 statistical trend, Figure 9I).

We found a positive correlation between B cells and monocytes in the percentage of intermediate (*r* = 0.413, *p* = 0.017; Figure 10A) and non-classical (*r* = 0.323, *p* = 0.067; Figure 10B) monocytes, intermediate (*r* = 0.433, *p* = 0.013; Figure 10C) and non-classical (*r* = 0.354, *p* = 0.051; Figure 10D) monocytes that express CD86, and the expression of CD86 in classical (*r* = 0.446, *p* = 0.010; Figure 10E) and intermediate (*r* = 0.371, *p* = 0.040; Figure 10F) monocytes.

There was a positive correlation between the number of NK cells and the percentage of non-classical monocytes that expressed CD86 (*r* = 0.349, *p* = 0.054; Figure 10G), the expression of CD86 in classical (*r* = 0.401, *p* = 0.023; Figure 10H), intermediate (*r* = 0.461, *p* = 0.009; Figure 10I), and non-classical (*r* = 0.469, *p* = 0.007; Figure 10J) monocytes, and the percentage of non-classical monocytes that expressed CD54 (*r* = 0.472, *p* = 0.009; Figure 10K).

Regarding the number of T-cytotoxic lymphocytes, we observed a positive correlation with the percentage of classical (*r* = 0.456, *p* = 0.013; Figure 11A) and non-classical (*r* = 0.453, *p* = 0.012; Figure 11B) monocytes that expressed CD54, and the expression of CD54 in classical (*r* = 0.351, *p* = 0.062; Figure 11C) and intermediate (*r* = 0.369, *p* = 0.049; Figure 11D) monocytes.

With respect to the relationship between lymphocytes and MVs, the percentage of T-helper lymphocytes was negatively correlated with the number (*r* = −0.500, *p* = 0.006; Figure 12A) and percentage (*r* = −0.364, *p* = 0.037; Figure 12B) of endothelial MVs, and positively correlated with the percentage of endothelial MVs (*r* = 0.588, *p* = 0.000; Figure 12C). CD4/CD8 was negatively correlated with the number (*r* = −0.429, *p* = 0.023; Figure 12D) and percentage (*r* = −0.588, *p* = 0.000; Figure 12E) of endothelial MVs.

Finally, the percentage of non-classical monocytes expressing CD86 was negatively correlated with the total number of MVs (*r* = −0.447, *p* = 0.025; Figure 13A). Moreover, there was a negative correlation between the expression of CD86 in classical monocytes and the percentage of endothelial MVs expressing tissue factor (*r* = −0.440, *p* = 0.025; Figure 13B). Of note, the expression of CD86 in intermediate (*r* = 0.378, *p* = 0.062; Figure 13C) and non-classical (*r* = 0.378, *p* = 0.057; Figure 13D) monocytes was positively correlated with the number of total MVs. There was a negative correlation between the percentage of intermediate monocytes expressing CD54 and the total number of MVs (*r* = −0.448, *p* = 0.028; Figure 13E) and endothelial MVs (*r* = −0.458, *p* = 0.037; Figure 13F).

## Discussion

In this cross-sectional study, we analyzed the immune phenotype of lymphocytes, monocytes, and MVs in patients with ACKD and KT vs. HS. The patients with KT showed B-cell lymphopenia, an increased proportion of T-cytotoxic lymphocytes, and increased levels of adhesion (CD54) and co-stimulatory (CD86) molecules in all monocyte subsets. Furthermore, the changes in lymphocyte subpopulations were positively correlated with the monocyte phenotypes, and both types of leukocytes were negatively correlated with changes in the MV phenotype. This is the first study that investigated the correlations between changes in lymphocytes, monocytes, and MVs. Although those changes could be directly or indirectly influenced by immunosuppressive treatment, the characteristic of those cells and molecules could participate in the development of cardiovascular and renal complications that persisted in patients with KT.

Currently, KT is the best therapy for CKD; however, patients require immunosuppressive treatment to avoid allograft rejection. The treatment may differ between patients due to numerous factors, such as the immunological risk for rejection, nutritional status, and the presence of other co-morbidities (51). Most patients with KT receive different immunosuppressive therapies that seek a balance to avoid acute rejection, toxicity (52), and possible deleterious effects, such as infections (53) and tumors (54). Most patients undergoing renal transplantation receive a combination (two or more) of calcineurin inhibitors (tacrolimus), azathioprine, mycophenolic acid, mammalian target of rapamycin (mTOR)-inhibitors, prednisone, and belatacept (51, 55–58).

The main objective of immunosuppressive treatment is the regulation of the T cell-mediated alloimmune response (51), which is induced by the response of the immune system to non-self-antigens of the same species. In this process, T cells play an essential role in recognizing the non-self-antigen in the context of the major histocompatibility complex (59, 60). Therefore, most immunosuppressors inhibited the activation of T cells and avoided the proliferation of activated B, T, and NK cells due to alteration in the synthesis of cytokines (61, 62). The present findings did not show changes in the total number of T lymphocytes and NK cells, whereas B-cell lymphopenia was noted in both groups of patients. Thus far, only a few studies have measured the total number of lymphocytes or the total number of T lymphocytes, without reporting any differences (63, 64). Other studies also showed the presence of B-cell lymphopenia in patients with ACKD (65, 66) and KT. Meanwhile, in ACKD, this diminution may be associated with a decrease in GFR. Nevertheless, in renal transplantation, the effect of treatment on GFR remains unclear. Some studies reported an increase in the number of NK cells (63); however, the changes in NK cells appeared to depend on the immunosuppressor treatment (64, 67).

Changes in the T-helper and T-cytotoxic subpopulations have been more widely investigated. The majority of the research studies, similar to the present investigation, did not report differences in the number of T subpopulations. Instead, they reported an increase in the proportion of the T-cytotoxic subpopulation compared with that of T-helper cells (63, 64). The regulation of T-helper cells may play a key role in the prevention of negatives outcomes in patients undergoing renal transplantation. Persistent CD4+ lymphopenia has been related to atherosclerosis (68) and an increase in morbidity and mortality in patients with KT (69).

Changes in monocyte subsets in renal transplantation have not been thoroughly studied. Intermediate (CD14++CD16+) and non-classical monocytes exhibited pro-inflammatory and proatherogenic activities (CD14+CD16+) in health individuals and in patients with CKD (4, 32, 70, 71). Some studies showed a depletion of non-classical monocytes due to treatment with glucocorticoids (72–74). The wide use of corticoids in immunosuppression may explain the decrease in non-classical monocytes recorded in the renal transplantation group.

There is limited research on the expression of CD86/B7.2 and CD54/ICAM1 in the monocyte subsets, particularly in CKD. CD80/b7.1 and CD86/B7.2 are co-stimulatory molecules, which are essential for the activation of T cells. This co-stimulation is exhibited by the antigen-presenting cells. Some studies did not report changes in the expression of CD86 in monocytes of patients with chronic renal failure (75), whereas others showed a decrease in its expression in monocytes (75) and dendritic cells (76) of patients undergoing dialysis. Nevertheless, the proinflammatory and proatherogenic monocytes showed an increase in CD86 expression (77, 78).

Although further research is warranted, the microinflammatory state of the CKD transplant could lead to the development of senescent monocytes with an increased expression of CD86, explaining the present results. Regarding the expression of CD86 by monocytes in patients with KT, the blockage of B7/CD28 co-stimulation required a specific antibody against B7 components (79–81). This is rarely used and had shown more significant effect but differs between the two subtypes of B7 due to differences in biochemical characteristics (82, 83). CD54/ICAM1 is an adhesion molecule expressed by immune and endothelial cells. The increased expression of ICAM1 in allograft tissue is related to rejection (84, 85). The monocytes of patients who underwent transplantation and were treated with mycophenolate mofetil did not show any differences in the expression of CD54 (86). The expression of CD86 and CD54 is markedly increased in intermediate and non-classical monocytes (4, 87, 88). These monocytes are highly proinflammatory and participate in atherosclerosis (4). The elevation in the expression of these molecules in all monocyte subsets of patients with transplantation may indicate an increase in senescent monocytes participating in cardiovascular disease, which is one of the main causes of death in patients with KT (89). The increase on the expression in costimulatory molecules has been shown in autoimmune disease; in particular, a increase of these costimulatory molecules in monocytes and in plasma lead to dysregulation of the immune response toward an exacerbate inflammatory one (90–92).

It was recently discovered that MVs are a form of extracellular communication. They play an essential role in the development of multiples disease (93, 94), but they have been extensively studied in cardiovascular alterations (95–98). In disease, there is an increase in the number and changes in the content of MVs (96). The increase in indoxyl sulfate shown in CKD has been related to the increase in endothelial MVs that participated in vascular calcification (98, 99). This increase in indoxyl sulfate and other uremic toxins may explain the increased number of MVs and endothelial MVs in patients with ACKD. Transplantation partially solves this problem by increasing kidney function. Tissue factor (CD142) triggers thrombotic responses and plays an important role in atherosclerosis. Thus, elevated levels of tissue factor in microparticles is associated with an increased risk of atherosclerosis and thrombosis (100–102). The elevation in the expression of tissue factor in patients with ACKD contributes to the increased risk of cardiovascular disease in patients with CKD.

To the best of our knowledge, this is the first study to correlate changes in lymphocyte subsets with different monocyte subtypes in renal transplantation. The cells of the immune system communicate through cytokines and microparticles to maintain the homeostasis of the organism. Monocytes influence T-cell differentiation by antigenic presentation, release of cytokines, or cell-cell communications (103). The present results showed the correlations of different phenotypes of lymphocytes with the three different subsets of monocytes and the expression of CD86 and CD54. Despite the renal transplantation, the leading cause of CKD and the co-morbidities persist.

Consequently, the microinflammation process continues, based on the persistence of the main cause of the disease and the alteration of renal alteration function (showed by a decreased GFR compared to with HS), which can modulate the different subsets of leukocytes in patients who undergo transplantation. Despite the immunosuppressive treatment, the monocytes are influenced by these effects. This leads to further alteration of the vascular endothelium, resulting in adverse cardiovascular outcomes. This is more important in the interaction between cytotoxic T-cells and endothelial MVs, leading to an increased risk of atherogenic complications in patients with transplantation.

Even though the promising results of this work, the vast variety of treatment, not only immunosuppression, but also concomitant medications such as statins and allopurinol, that CKD patients suffers complicates the study and analysis of these patients. Most of this concomitant medication has anti-inflammatory effects (104–107) and affected immune phenotypes (108–111). Also, said medication can change the number and content of MVs (112–114).

The main limitation of this study is the number of volunteer HS of the same socioeconomic status (2), which is an important factor influencing the outcome of the disease. Furthermore, the wide variety of immunosuppressive treatment options, as well as concomitant meditation and comorbidities, complicate the study of the effects of the drugs in monocytes and MV subsets. However, this study provides original and integrative knowledge regarding the differences and relationships of leukocyte subpopulations. This could lead to a better comprehension of the participation of the immune function in negative outcomes in patients who undergo transplantation.

In conclusion, B-cell lymphopenia and an increase in the expression of costimulatory and adhesion molecules were observed in patients with KT. These changes were interrelated and associated with the number of MVs. These findings can partially explain the negatives outcomes of cardiovascular disease in patients with renal transplantations and the persistence of adverse renal outcomes. Further prospective studies are warranted to elucidate this communication mechanism and its role in negative outcomes. The increase in risk factor linked to CKD and the high cost associated with renal substitutive therapies could bring a heavy burden to public healthcare systems in the near future.

## Data Availability Statement

The original contributions presented in the study are included in the article/supplementary material, further inquiries can be directed to the corresponding author/s.

## Ethics Statement

The studies involving human participants were reviewed and approved by Comité de ética de la investigación del Hospital Universitario 12 de Octubre. The patients/participants provided their written informed consent to participate in this study.

## Author Contributions

EM and JCarr conceived and designed the study. IG, CY, JCaro, and EM selected the patients and collected clinical and lymphocyte phenotype data. NC, GV, NS, CO, AF, and JCarr carried out the monocyte experiments. NC analyzed, collected monocyte data, carried out the graphical design, statistical analysis, and data interpretation. NS, AF, MA, RR, and JCarr performed the microvesicles experiments and analysis. NC and GV drafted the manuscript. MA, RR, EM, and JCarr edited and revised the manuscript. All authors contributed to the article and approved the submitted version.

## Funding

This study was supported by the Instituto de Salud Carlos III through the project PI17/01029, PI19/00240, and PI20/01321 (cofounded by the European Regional Development Fund A way to make Europe), and Sociedad Española de Nefrologia. NC was a fellow of the program Contratos Asociados a Proyectos de Investigación, Instituto de Investigación Sanitaria Hospital 12 de Octubre (imas12), Madrid, Spain. NS was a fellow of the program Becas Asociadas a Proyectos de Investigación, Instituto de Investigación Sanitaria Hospital 12 de Octubre (imas12), Madrid, Spain. AF was a fellow of the program Contratos Predoctorales de Investigación en Salud, Instituto de Salud Carlos III (FI20/00018). GV received a granted from the Comunidad de Madrid y Fondo Social Europeo (PEJ-2020-AI/BMD-18141).

## Conflict of Interest

The authors declare that the research was conducted in the absence of any commercial or financial relationships that could be construed as a potential conflict of interest.

## Publisher's Note

All claims expressed in this article are solely those of the authors and do not necessarily represent those of their affiliated organizations, or those of the publisher, the editors and the reviewers. Any product that may be evaluated in this article, or claim that may be made by its manufacturer, is not guaranteed or endorsed by the publisher.

## Abbreviations

ACKD: advanced chronic kidney disease
CKD: chronic kidney disease
GFR: glomerular filtration rate
HS: healthy subjects
MV: microvesicles
NK: natural killer.
